# Protein residue network analysis reveals fundamental properties of the human coagulation factor VIII

**DOI:** 10.1038/s41598-021-92201-3

**Published:** 2021-06-16

**Authors:** Tiago J. S. Lopes, Ricardo Rios, Tatiane Nogueira, Rodrigo F. Mello

**Affiliations:** 1 Department of Reproductive Biology, Center for Regenerative Medicine, National Center for Child Health and Development Research Institute, 2-10-1 Okura, Setagaya-ku, Tokyo, 157-8535 Japan; 2 Department of Computer Science, Federal University of Bahia, Salvador, Brazil; 3 Institute of Mathematics and Computer Science, University of São Paulo, São Paulo, Brazil; 4 Present Address: Itaú Unibanco, Av. Eng. Armando de Arruda Pereira, 707, Jabaquara, São Paulo 04309-010 Brazil

**Keywords:** Machine learning, Network topology, Protein analysis, Blood proteins, Haematological diseases

## Abstract

Hemophilia A is an X-linked inherited blood coagulation disorder caused by the production and circulation of defective coagulation factor VIII protein. People living with this condition receive either prophylaxis or on-demand treatment, and approximately 30% of patients develop inhibitor antibodies, a serious complication that limits treatment options. Although previous studies performed targeted mutations to identify important residues of FVIII, a detailed understanding of the role of each amino acid and their neighboring residues is still lacking. Here, we addressed this issue by creating a residue interaction network (RIN) where the nodes are the FVIII residues, and two nodes are connected if their corresponding residues are in close proximity in the FVIII protein structure. We studied the characteristics of all residues in this network and found important properties related to disease severity, interaction to other proteins and structural stability. Importantly, we found that the RIN-derived properties were in close agreement with in vitro and clinical reports, corroborating the observation that the patterns derived from this detailed map of the FVIII protein architecture accurately capture the biological properties of FVIII.

## Introduction

Blood coagulation is an elegant and efficient mechanism that starts immediately after a blood vessel is damaged, and results in the formation of a fibrin clot and a platelet plug that stops the bleeding. This process depends on the sequential activation of several coagulation factors, and the disruption of any of these steps leads to the impairment of this vital activity.

In this context, Hemophilia A (HA) is a coagulation disorder characterized by the presence of a defective version of the coagulation factor 8 gene. Depending on the type of mutation, it causes the synthesis of partially functional or non-functional FVIII protein, characterizing the severity of the HA symptoms^1^. The activated FVIII protein (FVIIIa) binds to the phospholipid membrane of activated platelets and serves as co-factor for the coagulation factor IXa, enhancing its activity more than 100,000 times^2^. Together, the FVIIIa-FIXa proteins form the so-called *tenase* complex, that converts the coagulation factor X (FX) to its active form FXa. In turn, FXa converts prothrombin to thrombin, already close to the final steps of the coagulation cascade^1,2^.

Previous studies determined the structure of FVIII (Refs.^3–5^), performed massive alanine mutagenesis experiments^6,7^, and made point mutations that increased the half-life of FVIII in circulation^5^. However, determining which residue substitutions are beneficial or detrimental to the FVIII activity remains a laborious and costly trial-and-error approach. Other groups used computational techniques to identify properties of the F8 gene and the FVIII protein that are related to the occurrence of mild or severe forms of HA (Refs.^8–13^). However, the limited input data and the lack of generalization to all residues precluded the understanding of the effect of substitutions of each specific residue.

In this study, we present a detailed map of the FVIII architecture with quantitative measures describing the role of each of its amino acids. We created a residue interaction network (RIN) of the FVIII protein in the form of a graph where the nodes are the ~ 1400 residues, and the edges represent interactions between these amino acids. Like other networks, this intuitive representation allowed us to calculate measures of centrality of each nodes, and to identify the hubs of the network (i.e., the nodes connected to several others and whose disruption leads the network to collapse). These approaches have been used extensively to study the robustness of electrical power grids^14^, transportation networks^15^, the influence of scientific papers^16^, and evidently, biological networks^17^.

In our case, the representation of the FVIII protein structure as a residues network and the study of its numerical properties helped us to identify residues responsible to maintain the structure in place, and to study the properties of the binding sites and their neighboring residues. Finally, we developed a machine learning framework that received as input the characteristics of this network and predicted the effect of targeted alanine mutations. The close agreement between the in silico, in vitro and clinical results demonstrate that it is feasible to capture and represent the biological properties of FVIII as a residue network.

## Results

### Construction of the network

**Figure 1:**
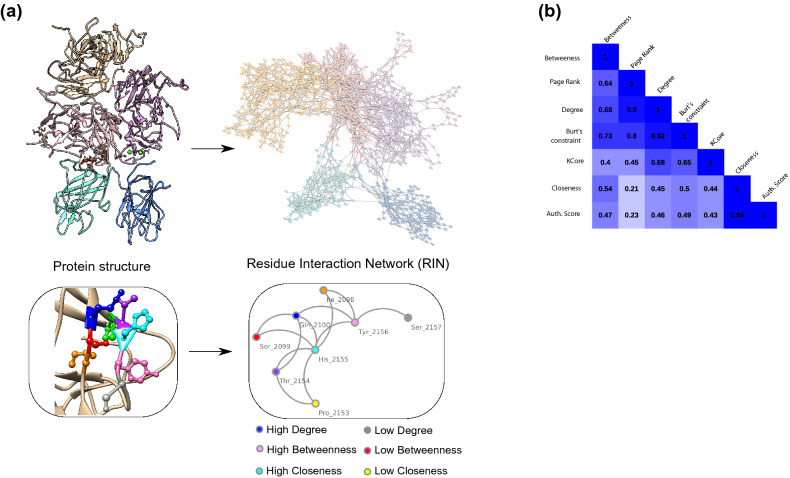
The FVIII Residue Interaction Network (RIN). (**a**) Each residue of the FVIII structure is represented as a node in the RIN. Two nodes are connected if either their main- or side-chains are close to each other (less than ~ 5 Å). Note that the RIN does not keep the three dimensional positional information of the domains or residues. The centrality of each node can be calculated based on the number of residues they interact (degree), whether they serve as bridges for groups of residues that would not be connected otherwise (betweenness), and are located in a position that requires few ‘steps’ to reach every other node in the network (closeness). Image created using the structure 2R7E (Ref.^3^) and Chimera 1.14 (Ref.^62^). (**b**) Several measures display a moderate to strong Pearson correlation to each other, despite being calculated using different underlying principles.

To create a RIN, we used all amino acids from the FVIII structure (PDB 2R7E Ref.^3^). as input to RINerator (Ref.^18^). This program follows three steps to create a RIN. First, it adds hydrogen atoms to the structure, which is essential to identify non-covalent interactions between amino acids^19^. Second, the non-covalent interactions are identified using a small probe (~ 0.25 Å) rolled around the van der Waals surface of each amino acid, and a contact is defined if the probe touches two non-covalently bonded atoms^20^. Finally, the interactions are summarized and the edges between nodes (i.e., amino acids) indicate that there is either a (i) side-chain–side-chain, (ii) side-chain–main-chain, (iii) main-chain–main-chain hydrogen bond or non-covalent interaction between the atoms of the residues (Fig. 1a). In the FVIII RIN, the distance between the residues’ atoms was less than ~ 5 Å, and we did not assign weights to the edges (Supplementary Table 1 contains the complete network).

In total, the FVIII RIN had 1336 nodes and 4074 edges. We did not attempt to fit its node degree distribution to a power-law because the layout of protein residue networks might change depending on the principles on which they are built^21^, and because scale-free networks are in fact very rare^22^.

Previous studies demonstrated that the centrality measures of amino acids in a RIN play an important role in the overall protein stability^21,23,24^, conformation^25^, and interaction with other proteins^26^. Therefore, to quantify the centrality of the FVIII RIN, we calculated 7 measures based on distinct underlying principles, namely, the degree, betweenness, closeness, Burt’s constraint, Page Rank-like, KCore, and the Authority Score (“Methods”).

We observed that these centrality measures were correlated (Fig. 1b), and we could avoid redundancy by using only three of them (degree, betweenness, and closeness). The degree and the betweenness are powerful measures to obtain *local* and *global* information about a node, i.e., the total number of neighbors a residue has, and the number of times a node serves as a bridge along the shortest path between two other nodes, respectively. The closeness has only global information, indicating from a given node, how many steps are necessary to reach every other node in the network^27^. The correlation we found indicate that although different measures quantified the centrality of amino acids from distinct perspectives, only three simple and well-studied measures were enough to appropriately describe the FVIII RIN.

Next, we wondered what was the relation between those centrality measures and the co-factor activity of FVIII. To answer this question, we used measurements of the FVIII chromogenic activity, expression and secretion. These measurements were obtained from massive mutagenesis experiments^6,7^ where almost all residues of the A2 and C2 domains were individually mutated to alanine, and the in vitro chromogenic activity and the secretion/expression levels (measuring thrombin formation and ELISA antigen binding, respectively). After close inspection of the distribution of the activity and secretion values of the mutant FVIII constructs, we divided the data into two groups, (i) low-chromogenic/low-expression (< 50% of wild-type), and (ii) high-chromogenic/high-expression (> 50% of wild-type).

**Figure 2:**
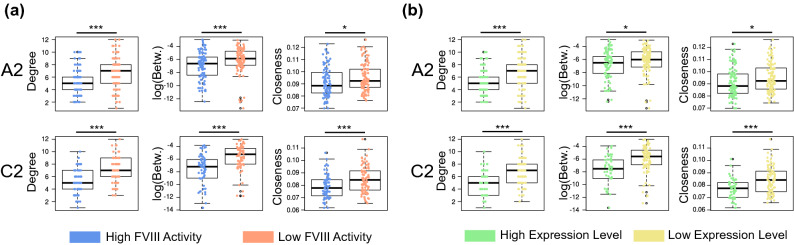
Centrality measures and mutagenesis results. (**a**) Alanine mutations on residues of the A2 and C2 domains that are more central in the FVIII RIN caused a reduction of the FVIII co-factor activity, measured by a chromogenic assay measuring thrombin formation^6,7^. (**b**) A similar effect is observed for the secretion and expression of the mutant constructs; here mutations at the central residues show a significant reduction in the expression/secretion levels, measured using the ELISA assay^6,7^. In all cases, the boxplots depict the median (center line), the first and third quartiles (lower- and upper-bounds), and 1.5 times the inter-quartile range (lower- and upper whiskers). Each dot in the plot is an amino acid mutation (i.e., an in vitro alanine mutant construct). Unpaired, two-sided Wilcoxon test (***Indicate p-values < 0.001; **p-value < 0.01; *p-value < 0.05).

We found that if mutated, the most central amino acids (i.e., the RIN nodes with high degree, betweenness and closeness values), caused a marked reduction or impairment of the FVIII secretion and co-factor activity (Fig. 2). Remarkably, this was consistently observed for both the A2 and the C2 domains even using different measures that express the centrality of amino acids. This overall pattern suggests that the FVIII mutant constructs that substituted the most central residues had a significant effect on the function of FVIII and produced proteins with the lowest expression levels^25,28^.

Taken together, these results demonstrate that the RIN representation of the FVIII protein is appropriate to study the activity of this coagulation factor. Moreover, the close agreement between in silico centrality measures and in vitro data is encouraging, because it allows us to quantify the importance of each individual residue of the FVIII structure.

### Machine learning framework predicts the in vitro chromogenic activity

Given that the alanine screening was performed for only two domains of FVIII (A2 and C2), we wondered if we could identify patterns in the RIN to predict the effect of alanine mutations in other domains.

For this purpose, we established a machine learning framework that received as input the network properties of the FVIII RIN, and a label indicating the chromogenic activity of the gene constructs containing an alanine mutation (i.e., High- or Low- activity).

**Figure 3:**
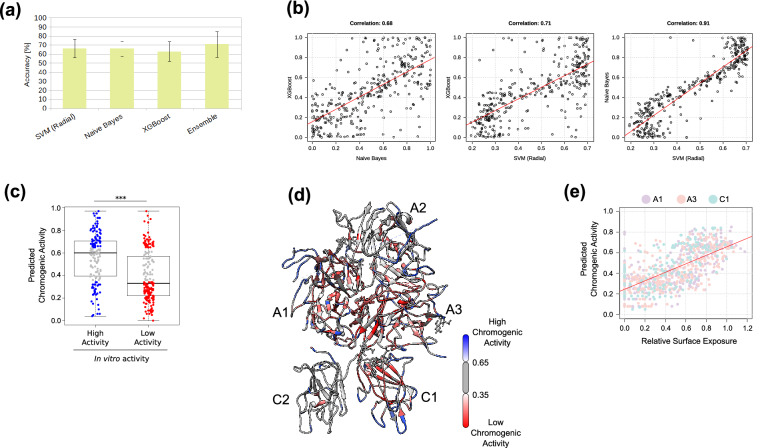
Machine learning framework and predictions. (**a**) The accuracy of both individual classifiers as well as the ensemble was calculated based on its correct classification of alanine mutations on the A2 and the C2 domains, using a 10 cross-fold validation (“Methods”). The variation in the accuracy values is due to the relatively small input (344 instances). The bars depict mean values and error bars, the standard deviation. (**b**) The predicted chromogenic activity outputted by the classifiers were only moderately correlated (Pearson’s correlation coefficient, p-value < 0.01). (**c**) In general, the FVIII mutant constructs with chromogenic activity similar to the wild-type form received high scores from the classifier ensemble. Likewise, low-activity mutants correctly received low scores. The boxplots depict the median (center line), the first and third quartiles (lower- and upper-bounds), and 1.5 times the inter-quartile range (lower- and upper whiskers). Each dot in the plot is an amino acid mutation (i.e., an in vitro alanine mutant construct). Unpaired, two-sided Wilcoxon test (***Indicate p-values < 0.001). (**d**) Predicted chromogenic activity mapped into the FVIII structure (Supplementary Table S2 lists all predicted values). (**e**) The relation between the predicted chromogenic activity and the relative surface exposure of the residues of the A1, A3, and C1 domains, indicating that perturbations to the ~ 10%–20% most buried residues (within the 0.1–0.2 range) will likely result in a considerable reduction of the chromogenic activity of the mutant construct. Each dot represents one amino acid from the A1, A3 and C1 domains. Image created using the structure 2R7E (Ref.^3^) and Chimera 1.14 (Ref.^62^).

We trained and evaluated 3 well-studied machine learning classifiers using this setup. Given the complexity of the problem and the small size of the input dataset (344 instances), we found that the individual classifiers performed well (Fig. 3a). However, upon close inspection of the results, we observed that the classifiers outputted different predictions for the same instances (Fig. 3b)—and this was the ideal setting for establishing an ensemble of classifiers^29^, in other words, combining the predictions of different algorithms to come closer to the real effect of alanine mutations.

The combination of classifiers using the median of the predicted probabilities considerably improved their predictive power, and by flagging mutations not clearly predicted as harmless or detrimental to the FVIII activity (Fig. 3c), we obtained an accuracy of over 70% (Fig. 3a).

We used this ensemble to predict the effect of alanine mutations at residues on the A1, A3 and C1 domains of FVIII. We observed that while most gene constructs containing an alanine mutation at the peripheral loop regions of FVIII were likely to retain the chromogenic activity similar to the wild-type, mutations at the buried core of the A1 and A3 domains were more likely to be harmful (Fig. 3d).

To quantitatively assess which residues were buried or exposed, we calculated the relative exposure of all amino acids by dividing the solvent-excluded surface area of the residue by the surface area of the same type of residue in a reference state; in our case, we used the reference values of the 20 standard amino acids in Gly-X-Gly tripeptides^30^. This normalization reduced the bias of classifying smaller residues as buried and larger residues as surface-exposed. We found an almost linear correlation between our predicted chromogenic activity and the relative exposure of the amino acids (Spearman Correlation 0.69, p-value < 0.001), suggesting that mutations at the 10% most buried residues are also likely to reduce the FVIII chromogenic activity to ~ 10% of its wild-type form (Fig. 3e).

Interestingly, we verified that among the FVIII positions with mutations predicted to be harmful, more than 67% had at least one form of HA reported in the EAHAD Hemophilia A mutation database^31^, against only 27% of the positions with mutations predicted to have chromogenic activity similar to the wild-type. This represents a significant association between the predicted chromogenic activities outputted by the machine learning framework, and the clinical symptoms caused by mutations on FVIII (p-value < 0.001, Fisher’s exact test. Supplementary Table 2 lists all predicted chromogenic activities).

In summary, these results indicate that a machine learning framework combined with the FVIII RIN successfully captured the in vitro chromogenic properties of FVIII. Importantly, we could generalize these findings to predict the effect of mutations observed in clinical settings (we report a complete characterization of the relation between the FVIII structure and clinical severity of HA in a separate study^32^).

### Identification of critical residues

After confirming the reliability of the RIN representation and its agreement with experimental mutagenesis data, we wanted to study the properties of residues that are important to maintain the FVIII structure in place. We termed them *critical residues*.

**Figure 4:**
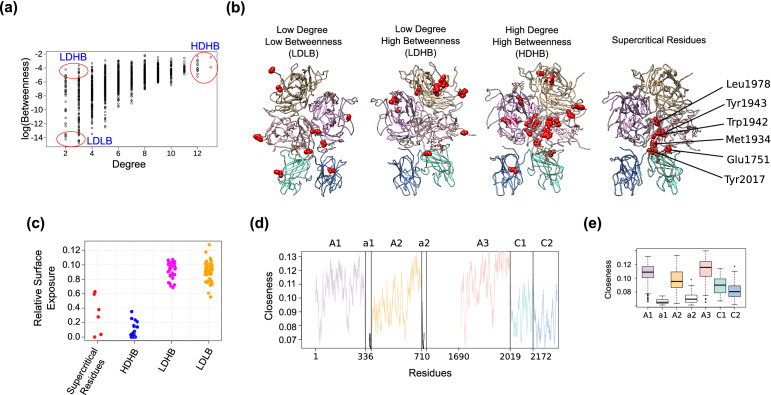
Critical residues in FVIII. (**a**) Depicted are the degree and betweenness of all residues of the FVIII RIN, and their assignment to groups that reflect their centrality characteristics. Each dot is one residue from the RIN. (**b**) The location of the different groups of residues on the FVIII structure. Images created using the structure 2R7E (Ref.^3^) and Chimera 1.14 (Ref.^62^). (**c**) The relative surface exposure of the key residues identified using the FVIII RIN centrality measures. (**d**) The closeness of each residue of the FVIII RIN, colored according to the domain where they are located. (**e**) Boxplot summarizing the closeness centrality of the residues of each FVIII domain. The boxplots depict the median (center line), the first and third quartiles (lower- and upper-bounds), and 1.5 times the inter-quartile range (lower- and upper whiskers).

For this purpose, we used two network centrality measures that are often studied together (degree and the betweenness), and defined three groups of residues, (i) high-degree and high-betweenness (HDHB), (ii) low-degree and high-betweenness (LDHB), and (iii) low-degree and low-betweenness (LDLB) (Fig. 4a).

We found that the HDHB residues have been conserved throughout evolution (as indicated by the ConsurfDB^33^ server), were buried at the core of FVIII and were either located at the interface of two domains or very close to it. For instance, Asp167, Arg1997, His2005, Leu2006, Met2010 are at the interface between the A1 and A3 domains, and Tyr656, Tyr664, Trp688, Trp1835 are located between the A2 and A3 domains. The LDHB residues were less conserved than their high-degree counterparts and located near or at one of the binding site of FIXa (e.g., Glu557, Arg562, Ser568, Asp712, Lys713). Finally, the LDLB amino acids were in general not conserved and located in the middle of small, sharp loops (e.g., Glu113, Ser1712, Ser1713, Phe2068, Asn2277) (Fig. 4b,c).

These findings indicate that the degree and the betweenness values accurately capture structural and conservation properties of the FVIII protein, including residues that ‘bridge’ different domains (HDHB), facilitate interaction to other proteins (LDHB), or serve as support and connector between the different protein parts (LDLB).

Next, after uncovering the properties of individual amino acids, we wanted to understand the connectivity characteristics of the domains of the FVIII protein. Using the closeness centrality, we found that while the C1 and C2 domains and the inter-domain regions a1 and a2 are the most peripheral parts of the FVIII protein, A3 is the most central domain, and compared to others, its amino acids are closer to all other residues in the protein structure (Fig. 4d,e). In biological terms, this suggests that the correct positioning of the A3 residues and their side chains is critical to maintain the long-distance communication (i.e., the allosteric communication network^26^) between residues located far from each other in the protein structure. Previous studies^34–36^ revealed that residues with elevated closeness values play a key role in the transfer of vibrational energy throughout the protein, stabilize its structure, induce conformational changes at distant sites and influence the protein function. Together, these findings point to potentially uncovered functional properties of the A3 domain.

Having identified the critical nodes of the FVIII RIN and A3 as the central domain, we asked which residues are the most central on the whole FVIII protein. To answer this question, we used all three centrality measures together (degree, betweenness, and closeness), and identified the residues that have molecular interactions with several other residues (high-degree), ‘bridge’ different parts of the protein (high-betweenness), and stabilize its overall conformation (high-closeness). We named them *super-critical* residues.

Using the Pareto front of the three centrality measures, we found that 6 residues had the highest values for all three centrality measures. These residues were highly conserved, deeply buried in the A3 core (Met1934, Trp1942, Tyr1943, Leu1978), or at the interface between the A3 and the C1 domains (Glu1751, Tyr2017) (Fig. 4b,c). Similar to the hubs of other biological networks^17^, disruptions at these sites propagate to the rest of the network, as evidenced by reports showing that the mutations Trp1942Arg (Trp1961Arg in the HGVS numbering) and Glu1751Lys (Glu1770Lys) cause conformational and functional changes associated to severe HA^37–40^.

Overall, these results indicate that centrality measures derived from the FVIII RIN can pinpoint specific residues and domains that are critical for the FVIII stability and function.

### Network properties of the FVIII binding sites

Seminal studies in the past 3 decades used a variety of molecular biology techniques to identify the binding sites of FVIII, and found that these residues are mainly organized in loops at the surface of the FVIII domains. Given that loops are known for their structural flexibility and motion, we wondered about their network connectivity, as well as the properties of the neighboring residues that hold the binding sites in place.

**Figure 5:**
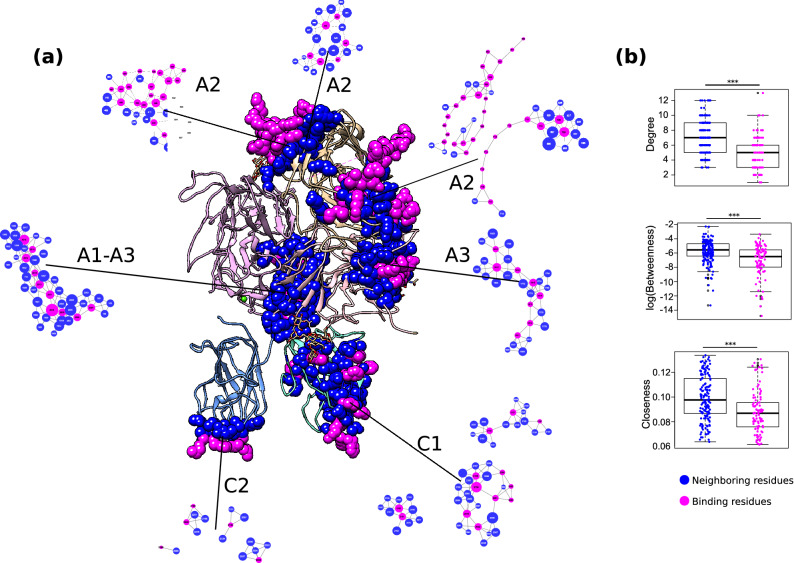
FVIII binding sites and neighboring residues. (**a**) Location of known FVIII binding sites (pink) and their immediate neighboring residues (blue) in the FVIII structure and in the RIN. Image created using the structure 2R7E (Ref.^3^) and Chimera 1.14 (Ref.^62^). (**b**) Comparison of the centrality measures of the residues reported to be part of a binding site and their immediate neighboring amino acids. The boxplots depict the median (center line), the first and third quartiles (lower- and upper-bounds), and 1.5 times the inter-quartile range (lower- and upper whiskers). Each dot in the plot is a node from the FVIII RIN (i.e., an amino acid). Unpaired, two-sided Wilcoxon test (***Indicate p-values < 0.001).

We used the FVIII RIN to identify the immediate neighboring residues of the binding sites interacting with FIXa (Refs.^41–45^), FX (Refs.^46–48^), thrombin^49,50^, von Willebrand Factor^45,51–57^, and the phospholipid membrane^51,53,54^. These binding sites were identified in the last 3 decades using site-directed mutagenesis, synthetic peptides, competition experiments, and detailed binding and enzyme kinetic assays (to this date, no complete FVIII structure in complex with other coagulation factors was determined). From these seminal studies, we identified 99 residues reported to participate in interactions with other coagulation factors (or with the phospholipid membrane), and 161 direct neighbors of those residues (Fig. 5a).

We found that both groups of residues formed tightly connected clusters where all amino acids were in close proximity to each other. While the residues reported to participate in protein interactions were not connected to numerous other residues, their immediate neighbors were significantly more connected (Fig. 5b), suggesting that the main- and side-chains of those neighboring residues are involved in multiple molecular interactions, creating a complex structure that holds the binding sites in place^25,28^.

This observation led us to speculate about the effects of residue substitutions at either the binding sites or their immediate neighbors. We searched the EAHAD HA mutation database^31^ and verified that while only 32% of the direct interaction sites had mutations associated to HA, 60% of their neighboring residues had cases reported, indicating a statistically significant association between mutations at neighboring residues of the interaction sites and the occurrence of Hemophilia A (p-value < 0.001, Fisher’s exact test).

These findings point to an emerging picture where the FVIII binding sites residues do not work in isolation; instead, they form together with their neighboring amino acids a delicate molecular network stabilized by multiple attractive and repulsive forces. In turn, due to its centrality measures higher than the residues of the binding site, the identity and proper positioning of the neighboring residues seems critical to the proper FVIII activity.

## Discussion

In the present study, we established a new representation of the FVIII structure and derived properties that quantify the importance of each of its residues. Our FVIII residue interaction network was created by representing amino acids as nodes, and connecting two nodes if the atoms of the main- or side-chains of two amino acids were in close proximity on the protein structure (Fig. 1). With this simple and intuitive representation, we identified the most central residues and verified that their centrality values matched the effects of in vitro mutations and amino acid substitutions associated to HA in patients.

The FVIII structure is held in place by a delicate yet precise set of molecular interactions. It is well-known that residues buried at the core of proteins are mainly hydrophobic, conserved, and that substitutions at these positions lead to impairment of the protein function^25,28^. However, for the vast majority of proteins—including FVIII—little is known beyond the hydrophobicity, charge, and surface exposure of amino acids. Therefore, we found that the RIN provided information about the neighborhood of all amino acids, and allowed us to mechanistically draw a map to understand why certain perturbations are more harmful than others.

Similar to other biological^17^ and non-biological networks (e.g., energy grids^14^ and transportation networks^15^), the central nodes are critical, and if perturbed, they are more likely to partially or completely disrupt the whole network. In proteins, this phenomena is starting to be mechanistically understood by the study of allosteric communication and regulatory networks, whereby the perturbation of certain residues cause conformational changes at distal parts of the protein^58^. Although the RIN does not directly address conformational changes to FVIII (which requires comprehensive molecular dynamics simulations), it paves the way for formulating hypothesis about the mechanism of changes that take place upon interaction with other proteins (e.g., conformation and orientation changes to FVIII itself^59^, as well as to its binding partners^60^).

Using the FVIII RIN, we observed that perturbations on the most central residues (in the form of targeted alanine mutations), caused a proportional loss in the secretion and chromogenic activity of FVIII (Fig. 2).

These quantifiable characteristics of residue importance enabled us to use a machine learning classifier to analyze all RIN properties in conjunction (Fig. 3), and subsequently we used the trained classifier algorithms to predict the chromogenic activities of hypothetical alanine mutations at residues from the A1, A3 and C1 domains (Supplementary Table 2). Interestingly, we found that amino acids at binding sites were not particularly central, creating the tempting hypothesis that they can be substituted in therapeutic proteins to increase the FVIII binding affinity and to modulate its immunogenic profile^61^.

Evidently, the residues at the binding sites of FVIII do not work in isolation (Fig. 5). As demonstrated in previous studies, binding sites residues and their immediate neighbors are organized in tightly connected modules^26^. These structures are relatively independent, and give rise to robustness against random mutations^26^; indeed, for FVIII it seems to be the case. The FVIIIa interaction partners (i.e., FIXa, FXa and vWF) bind to FVIII at multiple sites^2^, and although targeted mutations and competition assays with synthetic peptides reduced the affinity of the interactions^41–57^, they did not completely abolish them. Therefore, determining the effect of multiple mutations at different binding sites remains an interesting experiment to reveal the robustness limits of FVIII. In this sense, we are positive that the RIN presented here can be used to determine which residues should be mutated in conjunction.

In conclusion, our results demonstrate that the FVIII RIN captured the biological properties of FVIII, and effectively quantified and represented in silico the importance of each residue. While the FVIII RIN was constructed based on a ‘snapshot’ of the FVIII structure, it is a valuable resource to generate rational hypotheses to be tested experimentally, as well as to understand the mechanism of mutations causing pathological HA symptoms.

## Methods

### Database sanitation

We manually queried the European Association for Haemophilia and Allied Disorders Database (EAHAD) on 25^th^ June 2020. At present, the EAHAD is the largest source of information about hemophilia A mutation in the public domain. It is manually curated and contains both clinical and genetic information^31^. We selected 'Point' and 'Polymorphism' (on type), and 'Missense' (on variant effect) on the advanced search. It returned a total of 6,051 rows. Next, we removed mutations on the signal peptide regions, or outside the mature form of the protein, as well as instances with 1-st/2-st FVIII:C > 100. We also removed non-numerical values on the FVIII:C column, substituted the values > 5 for 5, < 10 for 10, < 11 for 11, " < 1" or " < 1" for "0". We also removed instances with FVIII:C values that would lead to ambiguous diagnostics (e.g., “0 to 2”, “ < 1 to 2”, < 2, etc.).

We substituted FVIII:C that contained ranges (e.g., "10 to 24") to the average value (in this example, 17). We removed instances without FVIII:C, and instances with discrepancies between "FVIII:C% (presumed 1-st)*" and "FVIII:C% (2-st/Chr)", and one mutation encoding a stop-codon.

Finally, we removed instances with ambiguous reported classifications (e.g., "mild/moderate", or "moderate/severe").

### Calculation of the FVIII protein structure properties

We used the FVIII protein structure deposited in the PDB with the accession 2R7E (Ref.^3^) and Chimera version 1.14 (Ref.^62^) to extract the solvent-excluded area (areaSES) and to calculate the relative surface exposure of all amino acids from this structure. We divided the solvent-excluded area of the residue by the surface area of the same type of residue in a reference state; in our case, we used the reference values of the 20 standard amino acids in Gly-X-Gly tripeptides^30^.

### The FVIII residue interaction network creation

We transformed the structure of the FVIII protein^3^ in an undirected, unweighted graph using RINerator version 0.5.1 (Ref.^18^) with the default parameters. We considered that two residues interacted if there was at least one edge between them, independently of the edge type. To analyze the RIN, we used R version 3.6.3 (https://www.R-project.org/) and the iGraph package, version 1.2.5 (Ref.^63^). With the iGraph package, we used the function *simplify* to remove redundant edges and self-interactions. Next, we calculated the degree, betweenness, closeness, Burt’s constraint^64^, Authority Score, Page Rank-like, KCore and the Authority Score measures.

We visualized the networks using Cytoscape version 3.8.2 (Ref.^65^).

Finally, we obtained the conservation score from the ConsurfDB webserver^33^, using the FVIII protein structure^3^ as input for search query.

### Classifier methodology

Supervised learning is a subarea of Machine Learning (ML) focused on producing the best possible mapping (model) $f:\chi \to  \Upsilon$ of examples ${x}_{i}$ in some input space $\chi$ to class labels ${y}_{i}$ in the output space $\Upsilon$. In the context of this work, input examples were composed of protein network centralities, and the class labels were the chromogenic activities of the mutant constructs (High: > 50% of wild-type, and Low: < 50% of wild-type).

In our experiments, we used the R statistical package 3.6.3 (www.r-project.org) and the MLR package (version 2.19.0) (Ref.^66^), which provides a unified interface to create machine learning models, and to perform training tasks such as hyperparameter tuning, cross validation, feature selection, ensemble construction, and results validation. Internally, the MLR package calls the e1071 package (version 1.4.1.1) (https://cran.r-project.org/web/packages/e1071/index.html last access: May 19, 2021) to create the SVM and Naive Bayes models and the xgboost package (version 1.7–6) (ref. ^67^) to create the ensemble method using the gradient boosting approach. All packages are available at the CRAN repository (https://cran.r-project.org).

### Experimental setup

The experimental setup of the machine learning framework followed the following steps: preprocessing, training and testing. We normalized all attributes to make sure our framework is not biased by data scales. We also removed all examples where values in at least one attribute was missing. We employed the tenfold cross validation method to reduce the chances of estimating overfitted models, and to ensure that the same sets of examples were considered by the different ML algorithms. This enabled a fair training and testing for all algorithms.

The training and test steps were performed using a grid search strategy to look for the best parametrization for all ML methods. The Support Vector Machine was assessed using the radial kernel according to a first empirical set of experiments: radial $\epsilon ^{{( - y[x - \omega ]^{2} )}}$. Given $\omega ,\mathrm{x}$ are two position vectors representing examples. For the radial kernel, we analyzed the following parameters $y=\{0.01, 0.02, \cdots , 1.5\}$.

The model obtained with Naïve Bayes has no parameter estimation.

Finally, the XGBoost model was estimated by running a grid search on the following parameters: maximum depth of a tree in {1, …, 25}, $y$ is the L_2_ regularization (Ridge Regression) term on weights in range [0, 1] to define the number of samples taken into consideration,$\eta \in [0,1]$ defining the learning rate by scaling the contribution of each tree, and obj is the loss function.

The best models obtained during the training phase with the tenfold cross validation strategy were chosen by their relative performances in terms of the Kappa index and the Area Under the ROC Curve (AUC). The Kappa index measures the agreement between the predicted and expected values, thus emphasizing that the results were not obtained by chance. This coefficient subtracts the expected from the observed agreement to quantify the probability of correct classifications by chance.

## Supplementary Information

Supplementary Legends.

Supplementary Table 1.

Supplementary Table 2.

## Data Availability

The code used in this study is available at https://github.com/ricardoarios/hemophilia-FVIII-RIN.

## Data availability

The datasets used in this study is available at https://github.com/ricardoarios/hemophilia-FVIII-RIN.
