# Supplementary material for: Protein residue network analysis reveals fundamental properties of the human coagulation factor VIII
Source: Sci Rep. 2021 Jun 16;11:12625. doi: 10.1038/s41598-021-92201-3 (PMC8209229; doi:10.1038/s41598-021-92201-3)
Supplement: Supplementary file 1 — Supplementary Legends. [file 41598_2021_92201_MOESM1_ESM.docx]

**Supplementary Tables**

**Supplementary Table 1 – Complete FVIII network and centrality measures.**

This table contains the complete FVIII RIN and the centrality measures derived from this network.

**Supplementary Table 2 – Predictions of chromogenic activity of alanine mutant gene constructs.**

This table contains the predicted chromogenic activities of residues located at the A1, A3 and C1 domains. These predictions were outputted by three classifier algorithms, as well as ensemble combining those values. Additionally, this table contains the relative surface exposure area of all RIN residues (Methods).
